# Supplementary material for: Changes in Physical Function, Cognitive Function, Mental Health, and Sleep Quality After Cardiac Surgeries and Procedures
Source: Nurs Rep. 2025 Jun 11;15(6):209. doi: 10.3390/nursrep15060209 (PMC12196282; doi:10.3390/nursrep15060209)
Supplement: Supplementary file 1 [file nursrep-15-00209-s001.zip › nursrep-3651965-supplementary.pdf]

Supplemental File

Table S1. Detailed scores in assessments

	Pre-operation	Post-operation	p-value
J-CHS*	2 (2–3)	3 (2–3)	< 0.01
Weight loss, n = 156, 156	47 (30)	52 (33)	0.54
Fatigue, n = 156, 156	35 (22)	49 (31)	0.07
Low physical activity, n = 156, 156	102 (65)	114 (73)	0.56
Slow walking speed, n = 155, 150	89 (57)	114 (76)	< 0.01
Muscle weakness, n = 155, 154	75 (48)	104 (68)	< 0.01
Grip strength, n = 155, 154	20.4 (16.5–27.3)	18.3 (14.5–23.9)	< 0.01
Barthel Index, n = 156, 156	95 (85–100)	75 (55–85)	< 0.01
Feeding	10 (10–10)	10 (10–10)	< 0.01
Moving back and forth between a wheelchair and bed	15 (15–15)	15 (10–15)	< 0.01
Grooming	5 (5–5)	5 (5–5)	< 0.01
Using a toilet	10 (10–10)	10 (5–10)	< 0.01
Bathing	5 (5–5)	0 (0–0)	< 0.01
Walking on a level surface	15 (10–15)	10 (5–15)	< 0.01
Moving up and down stairs	10 (0–10)	0 (0–0)	< 0.01
Dressing	10 (10–10)	10 (5–10)	< 0.01
Bowel continence	10 (10–10)	10 (5–10)	< 0.01
Bladder continence	10 (10–10)	10 (5–10)	< 0.01
HADS-A, n = 156, 156	3 (1–6)	1 (0–4)	< 0.01
I feel tense or ‘wound up’.	1 (1–2)	0 (0–1)	< 0.01
I get a sort of frightened feeling as if something awful is about to happen.	0 (0–1)	0 (0–1)	< 0.01
Worrying thoughts go through my mind.	1 (0–1)	0 (0–1)	< 0.01
I can sit at ease and feel relaxed.	0 (0–1)	0 (0–1)	0.89
I get a sort of frightened feeling like ‘butterflies’ in the stomach.	0 (0–1)	0 (0–0)	< 0.01
I feel restless as I have to be on the move.	0 (0–1)	0 (0–0)	0.15
I get sudden feelings of panic.	0 (0–1)	0 (0–0.3)	0.29

HADS-D, n = 156, 156	4 (1–7)	2 (1–6)	< 0.01
I still enjoy the things I used to enjoy.	0 (0–2)	0 (0–1)	0.04
I can laugh and see the funny side of things.	0 (0–1)	0 (0–0)	0.46
I feel cheerful.	0 (0–1)	0 (0–1)	0.38
I feel as if I am slowed down.	1 (0–1)	0.5 (0–1)	< 0.01
I have lost interest in my appearance.	0 (0–1)	0 (0–1)	0.03
I look forward with enjoyment to things.	0 (0–1)	0 (0–1)	0.31
I can enjoy a good book or radio or TV program.	0 (0–1)	0 (0–0)	0.50
MMSE, n = 156, 156	26 (24–29)	26 (24–29)	0.40
Orientation to time	5 (1–5)	5 (1–5)	0.15
Orientation to place	4.5 (4–5)	5 (4–5)	0.39
Memorization	3 (2–3)	3 (2–3)	0.03
Calculation	3 (1–5)	3 (1–5)	0.37
Recall	2 (2–3)	2 (2–3)	0.45
Naming	2 (2–2)	2 (2–2)	0.45
Repetition	1 (1–3.8)	1 (1–3)	0.80
Three-step command	3 (3–3)	3 (3–3)	0.09
Reading comprehension	1 (1–1)	1 (1–1)	0.18
Writing	1 (1–4)	1 (1–3.8)	0.09
Structure	1 (1–4)	1 (1–3.5)	0.87
Sleep quality, n = 156, 156	4 (3–5)	3 (2–4)	< 0.01

* In J-CHS, walking ability and grip strength tests were infeasible in some patients due to physical impairments.

J-CHS: Japanese version of the Cardiovascular Health Study; MMSE: Mini-Mental State Examination; HADS-A: Hospital Anxiety and Depression Scale for anxiety; HADS-D: Hospital Anxiety and Depression for depression
